# Protective Effect of Bergapten against Human Erythrocyte Hemolysis and Protein Denaturation *In Vitro*

**DOI:** 10.1155/2021/1279359

**Published:** 2021-12-21

**Authors:** Douglas Bosco Aidoo, Daniels Konja, Isaac Tabiri Henneh, Martins Ekor

**Affiliations:** ^1^Department of Pharmacology, Faculty of Pharmacy and Pharmaceutical Sciences, College of Health Sciences, Kwame Nkrumah University of Science & Technology, Kumasi, Ghana; ^2^Department of Biomedical Sciences, School of Allied Health Sciences, University of Cape Coast, Cape Coast, Ghana; ^3^Department of Pharmacotherapeutics and Pharmacy Practice, School of Pharmacy and Pharmaceutical Sciences, College of Health and Allied Sciences, University of Cape Coast, Cape Coast, Ghana; ^4^Department of Pharmacology, School of Medical Sciences, College of Health and Allied Sciences, University of Cape Coast, Cape Coast, Ghana

## Abstract

Bergapten, a furocoumarin found in many medicinal plants, is used for the management of various conditions. The present *in vitro* study evaluated the ability of bergapten to prevent human erythrocyte hemolysis and protein denaturation. Bergapten administered at 10, 30, and 100 *μ*g/ml exhibited a significant concentration-dependent protection on the erythrocyte membrane exposed to hypotonicity and heat-induced hemolysis. The concentration at which bergapten inhibited 50% of the cells from hemolysis (IC_50_) was determined on a dose-response curve, plotted as logarithmic (concentration) against percentage inhibition, keeping the hemolysis produced within the control group at 100%. Bergapten treatment produced an IC_50_ value of 7.71 ± 0.27 *μ*g/ml and 4.23 ± 0.42 *μ*g/ml for hypotonicity and heat-induced hemolysis, respectively. Diclofenac sodium at similar concentrations produced an IC_50_ value of 12.22 ± 0.30 *μ*g/ml and 9.44 ± 0.23 *μ*g/ml in the hypotonicity and heat-induced hemolysis, respectively. The ability of bergapten to inhibit protein denaturation was studied as part of an investigation on its mechanism of action. The results showed a significant concentration-dependent reduction in protein denaturation. When administered at 10, 30, and 100 *μ*g/ml, bergapten produced a concentration-dependent reduction in albumin denaturation. Bergapten inhibited protein denaturation with IC_50_ values of 5.34 ± 0.30 *μ*g/ml and 12.18 ± 0.20 *μ*g/ml in the heat-treated egg albumin and bovine serum albumin denaturation experiments, respectively. Diclofenac sodium (10, 30, and 100 *μ*g/ml) exhibited a similar protection against heat-treated egg albumin and bovine serum albumin denaturation experiments with IC_50_ values of 8.93 ± 0.17 *μ*g/ml and 12.72 ± 0.11 *μ*g/ml, respectively. Taken together, data from this study show that the pharmacological properties of bergapten may in part be related to its membrane-stabilizing and antidenaturation properties.

## 1. Introduction

The human red blood cells (RBCs), also referred to as erythrocytes, are the most abundant (˃80%) blood cells and vertebrates principal means of transporting oxygen from the lungs to the body tissues via the circulatory system. The mammalian RBC develops in the bone marrow, anucleate cells, and contains haemoglobin (a red iron-rich protein which gives the blood its characteristic colour) [1, 2]. In mature humans, RBCs are small, round, and biconcave but very flexible as they pass through extremely small blood vessels. The RBC membrane is a two-dimensional (2D) structure, comprised of a cytoskeleton and a lipid bilayer, tethered together via band-3 proteins at the spectrin-ankyrin binding sites and glycophorin at the actin junctional complexes [3]. Defects in the cytoskeleton membrane proteins compromise the integrity of the RBC, as seen in blood disorders such as sickle cell disease, haemolytic anaemia, thalassemia, spherocytosis, and elliptocytosis [4]. The lipid bilayer contains phospholipids, sphingolipids, cholesterol, and integral membrane proteins. Erythrocyte membranes perform similar functions to those of highly specialized cells in the body and are considered a simple cell model to study complicated biochemical phenomena [5]. Every small change in the architecture and composition of the erythrocyte membrane can affect the functioning of membrane protein ion and water channels which regulate the chemical and physiological balance in the cell. High polyunsaturated fatty acids (PUFAs) and the high cellular oxygen and haemoglobin (Hb) concentrations make erythrocyte membranes highly susceptible to oxidative damage [6]. Oxidants induce biophysical abnormalities in the erythrocyte membrane by decreasing cytoskeletal protein content and production of high molecular weight proteins [7, 8].

Studies have shown that agents that can stabilize the lysosomal membrane can prevent the release of phospholipase *A*_2_ from the liberation of arachidonic acid metabolites with its harmful effects [9, 10]. Also, the erythrocyte membrane is reported to be a structural analogue to that of the lysosomal membrane [11]; hence, agents that stabilize the erythrocyte membrane can be extrapolated to stabilize the lysosomal membrane and would serve as a good anti-inflammatory agent [12]. Activated neutrophilic lysosomal constituents, including bactericidal enzymes and proteases, are released upon lysosomal membrane damage. These activated constituents can cause further tissue damage after exposure to their extracellular environment [13]. As such, the inflammatory response can be controlled or limited by inhibiting the release of enzymatic lysosomal components by stabilizing the lysosomal membranes [14]. Additionally, denaturation of tissue proteins is one of the well-documented causes of inflammatory and arthritic diseases. Protein denaturation leads to the disruption of electrostatic, hydrogen, and disulphide bonds and the production of autoantigens in certain arthritic diseases [15]. The compounds which prevent these changes are known to exert potential antiarthritic activity.

Bergapten (5-methoxypsoralen), a furocoumarin found in medicinal plants, has become a compound of considerable interest in current research [16, 17]. Accumulated reports have demonstrated the potential therapeutic effects of bergapten in various disease conditions including psoriasis and vitiligo [18, 19], Alzheimer's disease (AD) [20], depression [21], osteoporosis [22], cancer [23], colitis [24], and asthma [25]. Prior *in vitro* studies on human peripheral blood mononuclear cells (PBMCs) showed that bergapten could suppress the release of lipopolysaccharide- (LPS-) stimulated proinflammatory cytokines such as tumour necrosis factor-alpha (TNF-*α*) and interleukin 6 (IL-6) production [26]. Furthermore, bergapten as reported by Zhou et al. [27] repressed the LPS activation of IL-1 *β*, prostaglandin *E* 2, nitric oxide (NO), and cyclooxygenase-2 (COX-2). An earlier report by Aidoo et al. [28] demonstrated that bergapten alleviates both compound 48/80, LPS and bovine serum albumin-mediated allergic hypersensitivity in both IgE-independent and IgE-dependent pathways of mast cell degranulation. Bergapten reduced cell infiltration into lung tissue and improved histological parameters (reduced oedema, congestion, and alveolar septa thickening) *in vivo*. The present study, which evaluated the ability of bergapten to stabilize the human erythrocyte membrane and inhibit protein denaturation, provides further insight into the mode of anti-inflammatory action of this compound.

## 2. Materials and Methods

### 2.1. Collection and Preparation of Erythrocyte Suspension

Fresh whole blood (3 ml) was collected intravenously from a healthy volunteer who had not taken any NSAIDs for two weeks prior to the experiment. The blood sample was collected into a heparinized vacutainer to prevent coagulation. Packed blood cells were obtained by washing the collected blood sample three times with 0.9% saline and centrifuged for 10 min at 3000 revolutions/minute. A stock solution of 10% *v*/*v* suspension of human red blood cells (HRBCs) was prepared using isotonic phosphate buffer (154 mM NaCl) in 10 mM sodium phosphate buffer (pH 7.4).

### 2.2. Preparation of Phosphate Buffer Saline (PBS)

A buffer solution was prepared using 800 g NaCl, 20 g of KCl, 144 g of Na_2_HPO_4_.2H_2_O, and 24 g of K_2_HPO_4_ in 8 L of distilled water.

### 2.3. Preparation of Hypotonic Solution (Saline Solution, 0.9% NaCl)

The saline solution was prepared by dissolving 9 g of NaCl in 700 ml of distilled water in a clean container. Water was added to make a 1000 ml volume to obtain 0.9% NaCl.

### 2.4. Drugs and Chemicals

Bergapten (5-methoxypsolaren) was purchased from Sigma-Aldrich (St Louis, USA). Bovine serum albumin (BSA) was purchased from PAA Laboratories (Marburg, Germany). Diclofenac sodium was acquired from Ernest Chemist (Accra, Ghana). All the chemicals used in this study were of analytical grade.

### 2.5. Membrane-Stabilizing Effect of Bergapten

#### 2.5.1. Hypotonic Solution-Induced Hemolysis

In this assay, the method described by Chandra et al. [29] was followed with slight modifications. Test samples consisted of 0.5 ml of HRBC stock mixed with 4.5 ml of hypotonic solution (0.9% NaCl) containing varying concentrations of bergapten (10, 30, and 100 *μ*g/ml). The positive control consisted of 0.5 ml of HRBC and 4.5 ml of hypotonic solution in varying concentrations (10, 30, and 100 *μ*g/ml) of diclofenac sodium (standard drug). The negative control sample consisted of 0.5 ml HRBC suspension mixed with 4.5 ml hypotonic solution alone. The experiment was carried out in triplicates. The mixture was incubated for 10 min at room temperature and then centrifuged for 10 min at 3000 rpm. The hemoglobin content of the supernatant was measured using a spectrophotometer (UVmini-1240, SHIMADZU) at 540 nm. The percentage inhibition of hemolysis was calculated using the formula

(1) $$\begin{array}{c} \%\text{ inhibition}=\left[\frac{\text{Absorbance}\left(\text{control}\right)-\text{Absorbance}\left(\text{test}\right)}{\text{Absorbance}\left(\text{control}\right)}\right]\times 100. \end{array}$$

#### 2.5.2. Heat-Induced Hemolysis

The method which had been previously described by Shinde et al. [30] and slightly modified by Henneh et al. [31] was followed. The reaction mixture (2 ml) consisted of 1.0 ml of 10% HRBC and 1.0 ml of various concentrations of bergapten (10, 30, and 100 *μ*g/ml) which was added to each tube and gently mixed. The positive control consisted of 1.0 ml of HRBC and 1.0 ml of various concentrations of diclofenac sodium (10, 30, 100 *μ*g/ml). The negative control consisted of 1.0 ml of 10% erythrocyte suspension and 1.0 ml of normal saline alone. The experiment was performed in triplicates. The resulting solution was heated at 56°C for 30 min and cooled to room temperature and centrifuged at 2500 rpm for 10 min. The supernatant was collected, and the absorbance of each solution was measured on a 560 nm (UVmini 1240, Shimadzu) spectrophotometer as an indicator of the degree of hemolysis. The percentage inhibition of hemolysis was calculated using the formula

(2) $$\begin{array}{c} \%\text{ inhibition}=\left[\frac{\text{Absorbance}\left(\text{control}\right)-\text{Absorbance}\left(\text{test}\right)}{\text{Absorbance}\left(\text{control}\right)}\right]\times 100. \end{array}$$

### 2.6. Protein Denaturation

#### 2.6.1. Egg Albumin Denaturation Assay

The method of Mizushima and Kobayashi [32] was followed with modifications by Obese et al. [33]. The reaction mixture (5 ml) consisted of 0.2 ml of fresh egg albumin, 2.8 ml of buffered phosphate saline (PBS, pH 6.4), and 2.0 ml of varying concentrations of bergapten (10, 30, and 100  *μ*g ml^−1^). The positive control consisted of 0.2 ml of fresh egg albumin, 2.8 ml of PBS (pH 6.4), and 2.0 ml of diclofenac sodium at varying concentrations (10, 30, and 100 *μ*g/ml). Negative control samples contained the same amount of egg albumin and PBS with 2.0 ml of distilled water. The mixture was incubated at 37 ± 2°C for 15 min and then heated at 70°C for 5 min to induce denaturation. After cooling, the absorbance was read at 660 nm (UVmini 1240, Shimadzu) using the vehicle as a blank. The experiment was carried out in triplicates. The percentage inhibition of protein denaturation was calculated using the formula

(3) $$\begin{array}{c} \%\text{ inhibition}=\left[\frac{\text{Absorbance}\left(\text{control}\right)-\text{Absorbance}\left(\text{test}\right)}{\text{Absorbance}\left(\text{control}\right)}\right]\times 100. \end{array}$$

#### 2.6.2. Bovine Serum Albumin Denaturation Assay

A previously described method by Leelaprakash and Dass [34] was used for the test. The reaction mixture consisted of 0.2 ml of 1% BSA and 2.8 ml of PBS (pH 6.4) added to 2.0 ml of normal saline (negative control), varying the concentration of bergapten or diclofenac sodium (10, 30, and 100 *μ*g/ml), respectively. The mixture was incubated at 37°C for 20 min and heated at 51°C for 20 min. The samples were cooled, and the absorbance was read at 660 nm using a spectrophotometer (UVmini 1240, Shimadzu). The experiment was carried out in triplicates, and the percentage inhibition was calculated using the formula

(4) $$\begin{array}{c} \%\text{ inhibition}=\left[\frac{\text{Absorbance}\left(\text{control}\right)-\text{Absorbance}\left(\text{test}\right)}{\text{Absorbance}\left(\text{control}\right)}\right]\times 100. \end{array}$$

### 2.7. Statistical Analysis

Data were analysed using GraphPad Prism for Windows Version 6.01 (GraphPad Prism Software, San Diego, USA). Results were presented as mean values ± standard error of mean (SEM), and statistical differences between treatment groups were compared using one-way analysis of variance (ANOVA) followed by Dunnett's *post hoc* test for multiple comparisons, with a 95% confidence interval. *P* < 0.05 was considered statistically significant.

## 3. Results

### 3.1. Membrane-Stabilizing Effect of Bergapten

#### 3.1.1. Hypotonicity-Induced Human Erythrocyte Membrane

**Figure 1:**
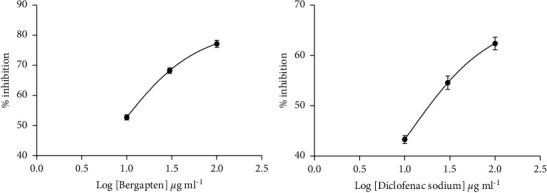
Dose-response curves for bergapten and diclofenac sodium in hypotonic solution-induced hemolysis. The negative control sample consisted of 0.5 ml HRBC suspension mixed with 4.5 ml hypotonic solution alone.

Bergapten showed a concentration-dependent anti-inflammatory activity and protected the human erythrocyte membrane exposed to the hypotonic solution. The concentration at which 50% of the blood cells were inhibited from hemolysis (IC_50_) was determined on a dose-response curve, plotted as log (concentration) against percentage inhibition, keeping the hemolysis produced within the control group at 100%. The IC_50_ value for bergapten was found at 7.71 ± 0.27 *μ*g/ml (Figure 1). Diclofenac sodium at the stated concentrations produced an IC_50_ value of 12.22 ± 0.30 *μ*g/ml (Figure 1).

#### 3.1.2. Heat-Induced Human Erythrocyte Membrane

**Figure 2:**
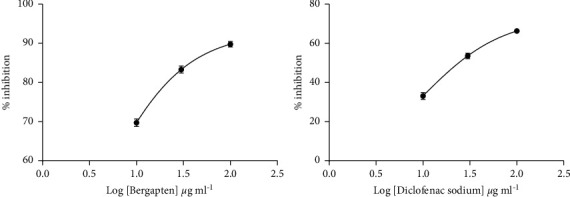
Dose-response curves for bergapten and diclofenac sodium in heat-induced hemolysis. The negative control consisted of 1.0 ml of 10% erythrocyte suspension and 1.0 ml of normal saline alone. Data were presented as mean ± standard error of mean (SEM).

When administered within a dose range of 10, 30, and 100 *μ*g ml^−1^, bergapten showed a concentration-dependent anti-inflammatory activity and protected the erythrocyte membrane exposed to heat. Bergapten concentration for 50% inhibition (IC_50_) was determined on a dose-response curve, plotted as log (concentration) against percentage inhibition, keeping the hemolysis produced within the control group at 100%. The IC_50_ value for bergapten was found at 4.23 ± 0.42 *μ*g/ml (Figure 2). A similar protection of 9.44 ± 0.23 *μ*g/ml was produced by diclofenac administration (Figure 2).

### 3.2. Protein Denaturation

#### 3.2.1. Effect of Bergapten on Egg Albumin Denaturation

**Figure 3:**
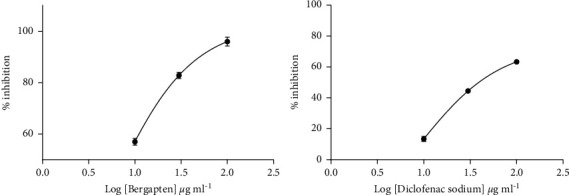
Dose-response curves for bergapten and diclofenac sodium in the egg albumin denaturation method. Data were presented as mean ± SEM.

Bergapten was investigated for its anti-inflammatory activity against heat-treated protein denaturation methods. Administered at 10, 30, and 100 *μ*g ml^−1^, bergapten showed a concentration-dependent reduction in albumin denaturation. Bergapten offered 50% inhibition (IC_50_) at 5.34 ± 0.30 *μ*g/ml to heat-treated egg albumin (Figure 3). Diclofenac sodium showed a similar protection to heat-treated egg denaturation with IC_50_ values of 8.93 ± 0.17 *μ*g/ml (Figure 3).

#### 3.2.2. Effect of Bergapten on the Denaturation of Bovine Serum Albumin

**Figure 4:**
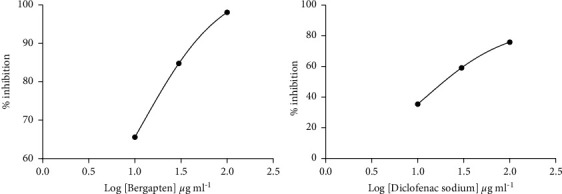
Dose-response curves for bergapten and diclofenac sodium in the bovine serum albumin denaturation method. Normal saline served as a negative control. Data were presented as mean ± SEM.

The heat-treated bovine serum albumin denaturation method was used to investigate the anti-inflammatory activity of bergapten. When administered at 10, 30, and 100 *μ*g ml^−1^, bergapten showed a concentration-dependent reduction in albumin denaturation. At a concentration 12.18 ± 0.20 *μ*g/ml, bergapten offered 50% inhibition (IC_50_) to heat-treated bovine albumin (Figure 4). Treatment with diclofenac sodium showed a similar protection to heat-treated bovine serum albumin denaturation with IC_50_ values of 12.72 ± 0.11 *μ*g/ml (Figure 4).

## 4. Discussion

In the screening of test agents for potential anti-inflammatory effect, membrane stabilization and the inhibitory effect on protein denaturation are of great interest because the vigor of every cell depends largely on the integrity of its membranes. Normally, an injury to the cell membranes makes the cell more susceptible to secondary damage through free-radical-induced lipid peroxidation [35]. Our aim was to investigate the potential stabilizing effect of bergapten on the human erythrocyte membrane (HRBC) exposed to hypotonic solution and heat-treated proteins as a possible mechanism of action of the compound. The erythrocyte membrane is well known to be a structural analogue to the lysosomal membrane [11]; hence, the stability of the erythrocyte membrane could be extrapolated to the stabilization of the lysosomal membrane [12].

From the study, bergapten (5-MOP) significantly protected the human erythrocyte membrane against hypotonic solution and heat-induced lysis. Bergapten produced IC_50_ values of 7.71 ± 0.27 g/ml for hypotonicity and 4.23 ± 0.42 *μ*g/ml for heat-induced hemolysis assays. On the other hand, diclofenac sodium had IC_50_ values of 12.22 ± 0.30 *μ*g/ml and 9.44 ± 0.23 *μ*g/ml in hypotonicity and heat-induced hypotonicity, respectively (Figures 1 and 2). Hypotonicity-induced hemolysis is the result of excessive fluid accumulation within red blood cells that causes rupturing of its membrane, causing secondary damage through free-radical-induced lipid peroxidation [36, 37]. Also, exposure of the erythrocyte membrane to excessive heat or hypotonic solution results in membrane lysis which is accompanied by oxidation of its haemoglobin [38]. Stabilization of the erythrocyte membranes, therefore, inhibits the rupture and subsequent release of activated neutrophil cytoplasmic components, including bactericidal enzymes and proteases that can exacerbate the inflammatory response after extracellular release [39]. Our findings suggest that bergapten might have produced its membrane stabilization property by increasing the surface area/volume ratio of cells or via its interaction with membrane proteins [40].

In addition, bergapten showed a concentration-dependent reduction in albumin denaturation when given at 10, 30, and 100 *μ*g/ml. Bergapten reduced protein denaturation in heat-treated egg albumin and bovine serum albumin with IC_50_ values of 5.34 ± 0.30 *μ*g/ml and 12.18 ± 0.20 *μ*g/ml, respectively. Diclofenac sodium (10, 30, and 100 *μ*g/ml), on the other hand, provided equivalent protection against heat-treated albumin denaturation with IC_50_ values of 8.93 ± 0.17 *μ*g/ml and 12.72 ± 0.11 *μ*g/ml, respectively. Protein denaturation assay was used as additional evidence for the membrane-stabilizing properties of bergapten. Proteins are denatured when they lose their secondary and tertiary structures through the application of external stress or compounds such as strong acids or bases. Many of the inflammatory disease conditions such as rheumatoid arthritis, serum sickness, glomerulonephritis, and systemic lupus erythematosus are normally associated with denaturation of tissue proteins. In arthritis, for example, the denaturation of tissue proteins leads to the production of autoantigens [15, 16]. Therefore, any agent that can prevent protein denaturation would be worth considering for anti-inflammatory drug development. Most nonsteroidal anti-inflammatory drugs (NSAIDs) are known to possess the intrinsic ability to stabilize or prevent heat-treated albumin denaturation at physiological pH 6.2–6.5 [41].

From our study, it can be seen that bergapten also dose-dependently prevented heat-treated egg albumin and bovine serum albumin denaturation, evidenced by the IC_50_ values obtained which were comparable to those obtained when diclofenac was used. Membrane proteins also control cell water/volume content by regulating the passage of ions such as sodium and potassium through ion channels. The inhibitory effects of bergapten on heat-treated proteins could be ascribed to the expansion and interaction with membrane proteins, an indication of antirheumatoid properties. Several clinical and experimental studies have shown that the pathophysiology of diseases such as malaria [42], diabetes [43, 44], artherosclerosis [45], sickle cell anaemia [46], and arthritis [15, 16] among others involve erythrocyte membrane instability coupled with protein denaturation. As such, the protective effects of bergapten against erythrocyte membrane destruction and protein denaturation could contribute to the amelioration of such diseases.

## 5. Conclusions

Taken together, data from this study show that the pharmacological properties of bergapten may, in part, be related to its membrane-stabilizing and antidenaturation properties. Bergapten no doubt shows promise as a drug candidate for managing inflammatory conditions and other pathologies associated with erythrocyte membrane instability or protein denaturation.

## Data Availability

All data generated or analysed during this study are included in this published article.

## References

[1] Kumar A., Verma M., Jha K. K. (2012). Resealed erythrocytes as a carrier for drug targeting: a review. *The Pharma innovation*.

[2] Jain T., Adhav R., Vaswani P. (2015). Erythrocytes as drug delivery system: a boon to cure. *International Research Journal of Pure and Applied Chemistry*.

[3] Li H., Lykotrafitis G. (2014). Erythrocyte membrane model with explicit description of the lipid bilayer and the spectrin network. *Biophysical Journal*.

[4] An X., Mohandas N. (2008). Disorders of red cell membrane. *British Journal of Haematology*.

[5] Nemkov T., Reisz J. A., Xia Y., Zimring J. C., D’Alessandro A. (2018). Red blood cells as an organ? how deep omics characterization of the most abundant cell in the human body highlights other systemic metabolic functions beyond oxygen transport. *Expert Review of Proteomics*.

[6] Hebbani A. V., Vaddi D. R., Padma Priya D. D., Varadacharyulu V. (2021). Protective effect of terminalia arjuna against alcohol induced oxidative damage of rat erythrocyte membranes. *Journal of Ayurveda and Integrative Medicine*.

[7] Suwalsky M., Orellana P., Avello M., Villena F. (2007). Protective effect of ugni molinae turcz against oxidative damage of human erythrocytes. *Food and Chemical Toxicology*.

[8] Yang H.-L., Chen S.-C., Chang N.-W. (2006). Protection from oxidative damage using bidens pilosa extracts in normal human erythrocytes. *Food and Chemical Toxicology*.

[9] Saffoon N., Uddin R., Subhan N., Hossain H., Reza H. M., Alam M. A. (2014). In vitro anti-oxidant activity and HPLC-DAD system based phenolic content analysis of codiaeum variegatum found in Bangladesh. *Advanced Pharmaceutical Bulletin*.

[10] Umukoro S., Ashorobi R. B. (2006). Evaluation of anti-inflammatory and membrane stabilizing property of aqueous leaf extract of momordica charantia in rats. *African Journal of Biomedical Research*.

[11] Chou C.-T. (1997). The antiinflammatory effect of an extract of tripterygium wilfordii hook F on adjuvant-induced paw oedema in rats and inflammatory mediators release. *Phytotherapy Research*.

[12] Amujoyegbe O. O., Agbedahunsi J. M., Akinpelu B. A., Oyedapo O. O. (2012). In vitro evaluation of membrane stabilizing activities of leaf and root extracts of calliandra portoricensis (JACQ) benth on sickle and normal human erythrocytes. *International Research Journal of Pharmacy and Pharmacology*.

[13] Vadivu R., Lakshmi K. S. (2008). In vitro and in vivo anti-inflammatory activity of leaves of symplocos cochinchinensis (lour) moore ssp laurina. *Bangladesh Journal of Pharmacology*.

[14] Omale J., Okafor P. N. (2008). Comparative antioxidant capacity, membrane stabilization, polyphenol composition and cytotoxicity of the leaf and stem of cissus multistriata. *African Journal of Biotechnology*.

[15] Duganath N., Rubesh Kumar S., Kumanan R., Jayaveera K. N. (2010). Activity of traditionally used medicinal plants. *International Journal of Pharma Bio Sciences*.

[16] Sun Y., Yang A. W. H., Lenon G. B., Yang A. W. H., Lenon G. B. (2020). Phytochemistry, ethnopharmacology, pharmacokinetics and toxicology of cnidium monnieri (L.) cusson. *International Journal of Molecular Sciences*.

[17] Pellizzeri V., Costa R., Grasso E., Dugo G. (2020). Valuable products from the flowers of lemon (citrus limon (L.) osbeck) and grapefruit (citrus paradisi macfad.) Italian trees. *Food and Bioproducts Processing*.

[18] Senol F. S., Woźniak K. S., Khan M. T. H. (2011). An in vitro and in silico approach to cholinesterase inhibitory and antioxidant effects of the methanol extract, furanocoumarin fraction, and major coumarins of angelica officinalis L. fruits. *Phytochemistry Letters*.

[19] Panno M. L., Giordano F. (2014). Effects of psoralens as anti-tumoral agents in breast cancer cells. *World Journal of Clinical Oncology*.

[20] Budzynska B., Skalicka-Wozniak K., Kruk-Slomka M., Wydrzynska-Kuzma M., Biala G. (2016). In vivo modulation of the behavioral effects of nicotine by the coumarins xanthotoxin, bergapten, and umbelliferone. *Psychopharmacology*.

[21] Ham J., Choi R.-Y., Lee H.-I., Lee M.-K. (2019). Methoxsalen and bergapten prevent diabetes-induced osteoporosis by the suppression of osteoclastogenic gene expression in mice. *International Journal of Molecular Sciences*.

[22] Inzinger M., Heschl B., Weger W. (2011). Efficacy of psoralen plus ultraviolet A therapy vs. biologics in moderate to severe chronic plaque psoriasis: retrospective data analysis of a patient registry. *British Journal of Dermatology*.

[23] Zhao Y., Wang N., Wu H. (2020). Structure-based tailoring of the first coumarins-specific bergaptol O-methyltransferase to synthesize bergapten for depigmentation disorder treatment. *Journal of Advanced Research*.

[24] Adakudugu E. A., Ameyaw E. O., Obese E. (2020). Protective effect of bergapten in acetic acid-induced colitis in rats. *Heliyon*.

[25] Adakudugu E. A., Obiri D. D., Ameyaw E. O. (2020). Bergapten modulates ovalbumin-induced asthma. *Scientific African*.

[26] Bose S. K., Dewanjee S., Sahu R., Dey S. P. (2011). Effect of bergapten from heracleum nepalenseroot on production of proinflammatory cytokines. *Natural Product Research*.

[27] Zhou Y., Wang J., Yang W. (2017). Bergapten prevents lipopolysaccharide-induced inflammation in RAW264.7 cells through suppressing JAK/STAT activation and ROS production and increases the survival rate of mice after LPS challenge. *International Immunopharmacology*.

[28] Aidoo, Douglas B., Obiri D. D. (2019). Allergic airway-induced hypersensitivity is attenuated by bergapten in murine models of inflammation. *Advances in Pharmacological Sciences*.

[29] Chandra S., Chatterjee P., Dey P., Bhattacharya S. (2012). Evaluation of in vitro anti-inflammatory activity of coffee against the denaturation of protein. *Asian Pacific Journal of Tropical Biomedicine*.

[30] Shinde U. A., Phadke A. S., Nair A. M., Mungantiwar A. A., Dikshit V. J., Saraf M. N. (1999). Membrane stabilizing activity—a possible mechanism of action for the anti-inflammatory activity of cedrus deodara wood oil. *Fitoterapia*.

[31] Henneh I. T., Ameyaw E. O., Biney R. P., Armah F. A., Obese E., Daniels K., Teye E. (2018). Ziziphus abyssinica hydro-ethanolic root bark extract attenuates acute inflammation possibly through membrane stabilization and inhibition of protein denaturation and neutrophil degranulation. *West African Journal of Pharmacy*.

[32] Mizushima Y., Kobayashi M. (1968). Interaction of anti‐inflammatory drugs with serum proteins, especially with some biologically active proteins. *Journal of Pharmacy and Pharmacology*.

[33] Obese E., Ameyaw E. O., Ameyaw E. (2018). Phytochemical screening and anti-inflammatory properties of the hydroethanolic leaf extract of calotropis procera (ait). R. Br. (apocynaceae). *Journal of Pharmaceutical Research International*.

[34] Leelaprakash G., Dass S. M. (2011). Invitro anti-inflammatory activity of methanol extract of enicostemma axillare. *International Journal of Drug Development and Research*.

[35] Halliwell B., Whiteman M. (2004). Measuring reactive species and oxidative damagein vivoand in cell culture: how should you do it and what do the results mean?. *British Journal of Pharmacology*.

[36] Augusto O., Kunze K. L., Ortiz de Montellano P. R. (1982). N-phenylprotoporphyrin IX formation in the hemoglobin-phenylhydrazine reaction. evidence for a protein-stabilized iron-phenyl intermediate. *Journal of Biological Chemistry*.

[37] Ferrali M., Signorini C., Ciccoli L., Comporti M. (1992). Iron release and membrane damage in erythrocytes exposed to oxidizing agents, phenylhydrazine, divicine and isouramil. *Biochemical Journal*.

[38] Ullah H. M., Zaman S., Juhara F. (2014). Evaluation of antinociceptive, in-vivo & in-vitro anti-inflammatory activity of ethanolic extract of curcuma zedoaria rhizome. *BMC Complementary and Alternative Medicine*.

[39] Kumar V., Bhat Z. A., Kumar D., Bohra P., Sheela S. (2011). In-vitro anti-inflammatory activity of leaf extracts of basella alba linn. var. alba. *International Journal of Drug Development & Research*.

[40] Guang-Ming Y., Wang D., Wei T. (2010). Anti-inflammatory and antioxidant activities of oxytropis falcata fractions and its possible anti-inflammatory mechanism. *Chinese Journal of Natural Medicines*.

[41] Hajare S. W., Chandra S., Sharma J., Tandan S. K., Lal J., Telang A. G. (2001). Anti-inflammatory activity of dalbergia sissoo leaves. *Fitoterapia*.

[42] Olanlokun J. O., Ekundayo M. T., Ebenezer O., Koorbanally N. A., Olorunsogo O. O. (2021). Antimalarial and erythrocyte membrane stability properties of globimetula braunii (engle van tiegh) growing on cocoa in plasmodium berghei-infected mice. *Infection and Drug Resistance*.

[43] Zhou Z., Mahdi A., Tratsiakovich Y. (2018). Erythrocytes from patients with type 2 diabetes induce endothelial dysfunction via arginase I. *Journal of the American College of Cardiology*.

[44] Foresto P., Arrigo M. D., Carreras L., Cuezzo R. E., Valverde J., Rasia R. (2000). Evaluation of red blood cell aggregation in diabetes by computarized image analysis. *Medicina-Buenos Aires*.

[45] Yamaguchi T., Ishimatu T. (2020). Effects of cholesterol on membrane stability of human erythrocytes. *Biological and Pharmaceutical Bulletin*.

[46] Oyenike M., Akpan H., Otulana O. (2019). In-vitro anti-sickling and membrane stability potentials of mishenland polyherbal extract on sickle red blood cells. *The Egyptian Journal of Haematology*.

